# Forces behind N- and C-capping of peptidic helices

**DOI:** 10.1039/d5cc04856g

**Published:** 2025-12-08

**Authors:** Tianxiong Mi, Lorenz Mattes, Thitima Pewklang, Karin Hauser, Kevin Burgess

**Affiliations:** a Department of Chemistry, Texas A & M University Box 30012 College Station Texas 77842 USA burgess@tamu.edu; b Department of Chemistry, University of Konstanz 78457 Konstanz Germany

## Abstract

Helical peptides are primarily stabilized by intramolecular hydrogen bonds. Particular conformational arrangements, capping motifs, help precisely terminate helices by compensating for disruption of helical H-bonding patterns. This contribution explores if: (i) N-and C-caps are essentially the same; and, (ii) how differences impact their thermodynamic helix stabilities and folding kinetics.

## Abstract 2

Helicity is more rigidly imposed in the N-capped system, and unwinding entropy is greater for the C-capped one.

## Graphical abstract

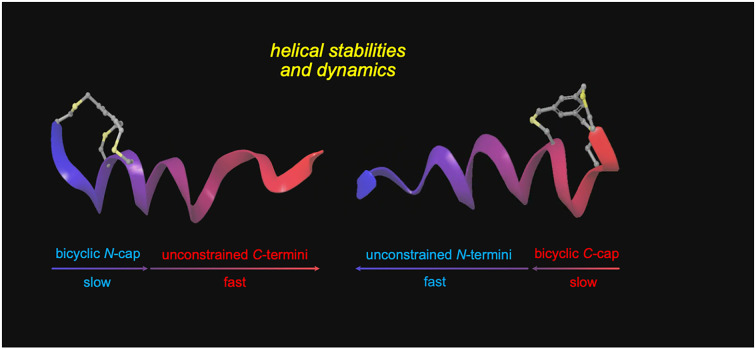

Formation of stable peptide helices is common,^1^ but still remarkable because subtle forces determine exactly where they begin and end.^2–4^ These forces must compensate for disruption of stacked *i* − *i* + 4 H-bonding, otherwise a set of H-bond donors would be unsatisfied at one terminus, and acceptors at the other. Evolution has solved this problem using ‘capping motifs’ to form alternative sets of hydrogen bonds, by introducing kinks which break the regular, helical *φ*, *ψ* dihedral angles (−60 ± 15 and −40 ± 15°). Thus, capped chains fold back on themselves to form H-bond patterns distinct from intra-helix ones. Casual readers might assume C- and N-capping motifs are essentially the same, but they are not. This contribution describes how we were motivated to look more closely at C- and N-capping motifs, and to use helical mimics to explore how differences impact stabilities and folding rates of helices stabilized by rigid, synthetic caps.

Recent work from one of our laboratories focused on design of peptidomimetics of natural N- and C-capping motifs.^5–7^ Bioinformatic studies performed on the entire Protein Data Bank at that time confirmed Schellman loops are prevalent C-capping motifs,^7^ and at N-termini dominant structures are ASX/ST motifs.^5^ However, data from our bioinformatic studies^5,7^ also revealed both capping motifs tend to feature triangulated clusters of lipophilic residues at well-defined orientations. Structures of these hydrophobic triangles are different in C- and N-caps. We realized both ‘hydrophobic triangles’ could be mimicked by a single benzenoid ring attached to three Cys side chains, appropriately spaced for C- and N-caps. Fig. 1a and b depicts Schellman loops and ASX motifs, and Fig. 1c and d shows peptidomimetics we designed to mimic them. Both designs are conceptually unique, more rigid and accessible than any in the literature preceding them. Prior work by others had generated several cap designs for peptide N-termini, but none of note for C-termini.

**Fig. 1:**
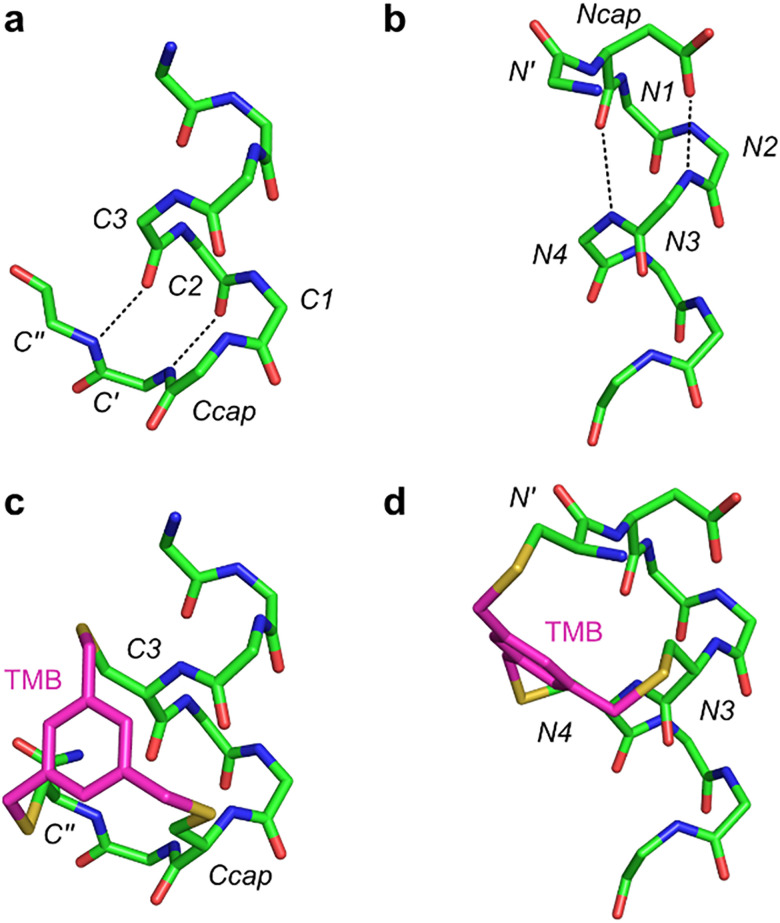
Structures of: typical Schellman (a) and ASX (b) motifs, and of our C-cap (c) and N-cap designs (d). TMB refers to 1,3,5-trimethylbenzene.

Our previous work on these bicyclic cap systems focused on developing a biomimetic and experimentally convenient approach to prepare robust helical mimics. However, it opened a unique opportunity to study entirely different phenomena: how these capping systems could report on the kinetics of helical folding. It was foreseen this might be possible by comparing a peptide analog bearing a rigid C-cap with a similar one containing an N-cap. Our objectives were to determine differences in the thermodynamic stabilities and unfolding kinetics of these systems, and to correlate them to their structures.

*Design of test systems*. First, we set out to identify a suitable number of amino acids to use for all four compounds. For the linear, unconstrained peptide, L, random coil conformations should prevail so this could serve as a negative control, but it had to be designed carefully. If too many amino acids were used then that control would show some helicity. Further, we wanted analogs with the same number of amino acids but this caused an opposing limitation: if our systems contained too few residues the dual capped system (dual D, *i.e.* with C- and N-caps) would be too short to accommodate N- and C-caps and at least one or two helical turns of unconstrained amino acids. Thus a suitable compromise had to be found for L and D to serve as negative and positive controls for helical genesis and stabilization for the key compounds, the mono-capped analogues (N, N-capped, and C, C-capped).

Two additional criteria were set in selecting compounds to study. First, they should contain Trp (W) so their concentrations could be measured by UV, and normalized to ensure uniformity in key experiments. Second, they were based on repeating –KAAA– sequences giving systems with minimal complicating side-chain effects (hence Ala, A, rich) and have water solubility (hence contain K, Lys), just as others have in the field before us.^8–10^

Based on the discussion above, dual-capped: D15 (W{CDAACC}_bicyclo_AK{CAACGC}_bicyclo_); D17 (W{CDAACC}_bicyclo_AKAA{CAACGC}_bicyclo_); and, D22 (W{CDAACC}_bicyclo_AKAAAKAAA{CAACGC}_bicyclo_), were synthesized to establish the minimum number of amino acids required. CD spectra (Fig. 2a, 298 K, PBS) revealed a distorted, noncanonical signature for D15 (226/205 ellipticity ratio ∼1.7), so this one was too short. However, D17 (ellipticity ratio ∼1.0) and D22 (∼1.0) had a CD spectrum characteristic of helical systems. Encouragingly, L17 (acetyl-AKAAAAKAAAAKAAAAW) was not helical so could serve as the negative control. The rest of the study was therefore narrowed to comparison of the key compounds: C17 (acetyl-AAAAKAAAAKW{CAACGC}_bicyclo_); and, N17 (W{CDAACC}_bicyclo_AAAKAAAAKA) against the random coil and helical extremes, L17 and D17.

**Fig. 2:**
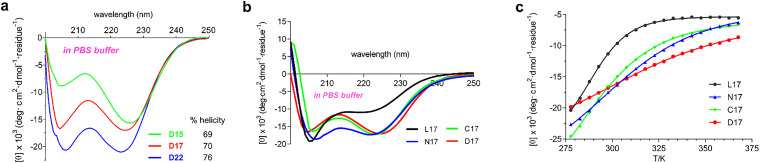
(a) CD spectra of three dual capped peptides with two, four, and nine cap-spanning residues, respectively (10 µM in PBS buffer at 298 K). (b) CD spectra of L17 (black throughout), N17 (blue), C17 (green), and D17 (red; all 10 µM at 25 °C in pH 7.4 PBS). L17 exhibited minimal helical content throughout. (c) Temperature dependence of mean residue ellipticities at 222 nm from 277.6 to 367.0 K.

*CD studies to explore thermodynamics of the folded states*. CD studies on the 17-mer systems reflect thermodynamic parameters for helical conformations (Fig. 2b, c and Table 1) which follow the trend: L17 < N17 ∼ C17 < D17. At 298 K, C17, N17, and D17, exhibited greater helicity than L17, *ie* higher 222 nm ellipticities, and 222/206 ellipticity ratios near unity, both signatures of α-helical shapes.

**Table 1:** Unfolding thermodynamic parameters of the featured peptides based on Van’t Hoff analyses

Peptide	Helicity@298 K^a^ (%)	Δ*G*_298K_ (J mol^−1^)	*T*_m_ (K)	Δ*H* (J mol^−1^)	Δ*S*_*T*_m__ (J mol^−1^ K^−1^)
L17	34	−496.6	288.9	16400	56.7
C17	60	−365.8	287.6	9220	32.1
N17	65	121.8	302.4	7840	25.9
D17	70	238.8	310.6	6020	19.4

a Calculated using.^8^

CD spectra were also used to compare linear, N, C-, and dual-capped mimics in our original report of dual capped systems.^6^ Those mimics were each of different lengths, and based on the –IRESLL– sequence flanked by different residues to correspond to mimicry of a particular protein. Consequently, those structures and most of the data collected were inappropriate for this study. However, per residue helicities in that series correspond to the test compounds here: linear peptide control < N-cap ∼ C-cap ≪ dual cap (C and N). This indicates the trends observed for L, N, C, D here are not specific to a particular amino acid sequence, and variations introduced by using more diverse residues have less impact than capping constraints.

Next, CD spectra variations with temperature were explored. VT-CD revealed distinct unfolding profiles for the four peptides. Unfolding was quantified using Van’t Hoff analyses, based on changes in ellipticity at 222 nm assuming a two-state transition, as previously described.^11^ Fitting methods are described in the SI. At 298 K, L17 exhibited the most negative free energy, indicating the greatest tendency to unfold. Median transition temperatures (*T*_m_'s) increased in the order: L17 (288.9 K) ∼ C17 (287.6), N17 (302.4), D17 (310.6). L17 displayed the largest unfolding enthalpy and entropy, while introduction of caps significantly reduced both, suggesting structural differences between folded and unfolded states progressively narrow: L17 > C17 > N17 > D17.

*NMR Studies Of Solution Conformations.* Two insurmountable problems were encountered when NMR experiments were attempted on 17-mer peptides. First, they contain many alanine residues (10/17 for C17 and N17, and 13/17 for L17), hence there was severe CαH region overlap. Second, aggregation broadened the peaks at higher concentrations. Ultimately, we were unable to make enough unambiguous assignments for interpretation. However, another possibility emerged, as below.

Extensive NMR data was collected to explore the helicity of 12-mer analogs, C12 (acetyl-AAAAKW{CAACGC}_bicyclo_) and N12 (W{CDAACC}_bicyclo_AAAKA), in the original work on these capped systems.^5,7^ This published data was reconsidered with a different objective than establishing overall helicity, *ie* to assess differences between C12 and N12, and two were found. ^3^*J*_NH–αH_ of ideal α-helical peptides is 3.9 Hz based on Karplus' equation.^12^^3^*J*_NH–αH_ coupling constants for residues following the cap are smaller and closer to ideal (3.9 Hz) for helices in N12 (four residues after the bicyclic cap: 5.2, 5.5, 5.3, 5.2) compared to C12 (5.2, 5.7, 5.4, 5.9). Furthermore, bicyclic cap in N12 induces distinct diastereotopic CβH_2_ protons in three Cys (C1, C2, C3), whereas in C12 this is observed for only two (C2, C3). These two subtle but measurable differences imply the bicyclic N-cap exerts a stronger helix-ordering effect and imposes greater rigidity than the C-cap.

*Temperature-jump IR to explore kinetics*.^13^ Peptides and peptidomimetics in solution (5 mg mL^−1^ in D_2_O) were allowed to reach equilibrium at controlled temperatures *T*. Intense nanosecond 2090 nm laser pulses were applied to induce rapid temperature jumps, after which the systems relax to new equilibria at the elevated temperature. Transitions between those two equilibrium states were monitored by changes in amide-I band absorption at ∼1630 cm^−1^. Thus unfolding from more to less helical states were observed. Two key parameters for the transitions were: time constants *τ* (ns), and absorbance changes *A* (%). Time constants reflect unfolding kinetics (*τ* = 1/rate constant), and absorbance changes indicate IR absorbance variations with structural changes.

Observations *via* IR require higher concentrations than CD spectroscopy. Insufficient water solubility of D17 meant samples of this were made in 20% DMSO/D_2_O (20 mg mL^−1^). Absorbance changes for D17 were relatively small, even at that concentration, hence the data gives larger errors. We believe the absorbances changes were small because D17 is predominantly helical at the two temperatures studied: 298 and 307 K, so there are few structural and IR changes to observe.

Kinetic data for negative and positive controls L17 and D17 fitted monoexponentially. For D17, this transition involved a minor conformational change almost not detectable (Δ*A* < 0.1 mOD at 297 K) because both are helical, as asserted above. For L17 the absorbance change was large (Δ*A* = 1.5 mOD at 298 K), presumably because the peptide lost helicity and became random coil as temperature increased.

Contrary to data for the controls, key compounds N17 and C17 followed biexponential fits: one fast “*τ*_f_” (270 and 255 ns, respectively) and another slower “*τ*_s_” (4.75 and 4.05 µs; all at 298 K). The fast transitions (*τ*_f_, ∼ hundreds of nanoseconds) occurred with large amplitude fractions (*A*_f_, 64% for N17 and 85% for C17) dominating the relaxation dynamics whereas the slow ones (*τ*_s_, single digit microseconds) contributed much less (*A*_s_ = 36% for N17 and 15% for C17).

Time constants of hundreds of nanoseconds are typical for α-helical peptide folding dynamics.^4,14–17^ Fast time constants, *τ*_f_, of that magnitude were observed for L17, N17, and C17. Our interpretation is slower rates, higher *τ*_s_, for N17 and C17 are related to residues within and/or near the rigid caps. Amplitude fractions *A*_f_ and *A*_s_ are consistent with this interpretation. Helix nucleation from the synthetic caps imposes diminishing kinetic ordering effects with distance from the constrained termini. Slow steps are associated with lower absorption change fractions because regions proximal to the caps are more rigid and resistant to unfolding, resulting in smaller conformational changes upon increasing temperature. Detailed analyses of the kinetic data will be presented elsewhere, but the salient features are as follow.

This study was performed to focus on differences between the N- and C-capped systems, interpreted relative to L and D controls. N17 and C17 had distinct rate variations with temperature. Time constants *τ*_f_ and *τ*_s_ ∼298 and 307 K were about the same for N17 (270 ± 81, 240 ± 170; and, 4750 ± 1200, 3850 ± 770 ns, respectively). Conversely, C17 unfolded *faster* at the higher temperature (*τ*_f_: 255 ± 140, 113 ± 73; and, *τ*_s_: 4050 ± 2300, 3350 ± 620 ns). These differences between N17 and C17 are small, but experimentally significant.

*Unified interpretation of thermodynamic and kinetic data*. Kinetic data shows equilibria changes between higher and lower populated helicity on temperature jump is greater for C17 than N17. Thermodynamic parameters deduced from VT-CD show Δ*S*_N17_ < Δ*S*_C17_; which is simply a different way of expressing the same observation. Thus, the order change from predominantly helical to random coil is greater for the C-capped system.

Greater order change from predominantly helical to random coil for the C-capped system may be interpreted by referring to Fig. 3. N-Capped systems feature two constraints to intra-helix residues N3 and N4, whereas the C-capped mimic only has one, to C3. Thus helicity is more rigidly imposed in the N-capped system. Our C- and N-capped mimic designs are based on Schellman loops and ASX/ST motifs, respectively, so exactly the same conclusions should apply to these natural systems in cases where all extraneous factors are equal. Thus, N-capped systems, ASX/ST motifs, are more stringent enforcers of helicity than Schellman loops, the C-capped ones. At higher temperatures, when the uncapped residues presumably are completely non-helical, the structure of the N-cap, having two constraints, will still bias the first four residues towards the first turn of a helix, more than the C-cap does towards the last. This correlates with the positions of Cys alkylations in these mimics because, recall, those positions were designed to mimic the distinct hydrophobic channels at the C-terminus. Similar forces are still imposed for Schellman and ASX/ST systems by their H-bond patterns and hydrophobic triangles.

**Fig. 3:**
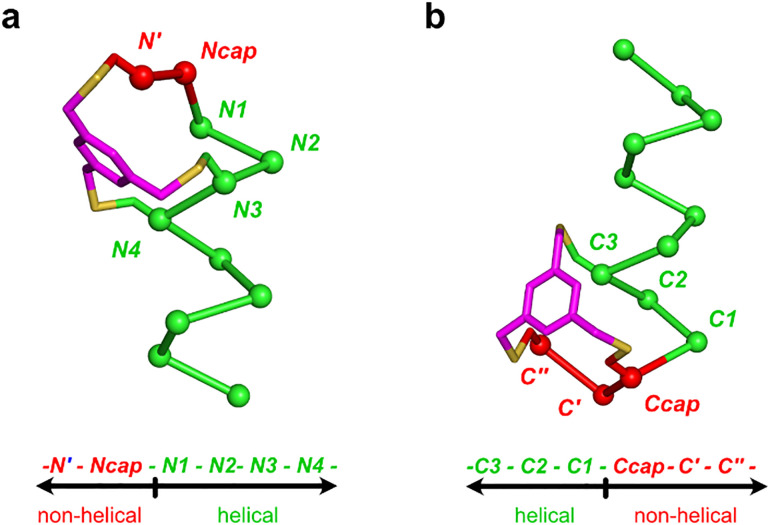
N-Cap peptidomimetic (a) includes two intra-helix constraints (N3, N4) and four helical residues (N1–N4), while C-cap (b) only has one (C3) and three (C3–C1), respectively.

The affects postulated above are subtle, and may well be obscured or accentuated by other differences in specific peptide or proteins systems. However, we suggest these energetic biases may apply across the whole proteome, and will become apparent when adjusted for random, case-by-case differences. This is important because these small differences can influence helical genesis and stabilization in tens of thousands of protein and peptide systems.

## Conflicts of interest

The authors declare no competing interest.

## Supplementary Material

CC-062-D5CC04856G-s001

## Data Availability

The data supporting this article have been included as part of the supplementary information (SI). Supplementary information: peptide syntheses and characterization; and, experimental details for: CD and IR-detected temperature jump studies with equations for data interpretation. See DOI: https://doi.org/10.1039/d5cc04856g.
