# Correction: Forces behind N- and C-capping of peptidic helices

**DOI:** 10.1039/d6cc90257j

**Published:** 2026-08-21

**Authors:** Tianxiong Mi, Lorenz Mattes, Thitima Pewklang, Karin Hauser, Kevin Burgess

**Affiliations:** a Department of Chemistry, Texas A & M University, Box 30012 College Station Texas 77842 USA burgess@tamu.edu; b Department of Chemistry, University of Konstanz Konstanz 78457 Germany

## Abstract

Correction for ‘Forces behind N- and C-capping of peptidic helices’ by Tianxiong Mi *et al., Chem. Commun.*, 2026, **62**, 3028–3031, https://doi.org/10.1039/d5cc04856g.

The authors regret that the acknowledgements with the funding information were omitted in the original article. The following acknowledgements paragraph should have been included:

“Financial support for this project was provided by NIH R21NS130471 (supplement 1R21NS130471-01A1), the Welch Foundation AU-2182-20240404, the Texas A&M University T3-Grants Program (246292-00000), and CPRIT RP250550.”

The Royal Society of Chemistry apologises for these errors and any consequent inconvenience to authors and readers.

