# Supplementary material for: Forces behind N- and C-capping of peptidic helices
Source: Chem Commun (Camb). 2025 Dec 8;62(9):3028–31. doi: 10.1039/d5cc04856g (PMC12801117; doi:10.1039/d5cc04856g)
Supplement: CC-062-D5CC04856G-s001 [file CC-062-D5CC04856G-s001.pdf]

Supplementary Information (SI) for ChemComm.
This journal is © The Royal Society of Chemistry 2025

Electronic Supplementary Information

Forces Behind *N*- And *C*-Capping of Peptide Helices

Tianxiong Mi[a], Lorenz Mattes[b], Thitima Pewklang[a], Karin Hauser[b] and Kevin Burgess[a]*

[a]*Department of Chemistry, Texas A & M University, Box 30012, College Station, Texas 77842, USA.*

[b]*Department of Chemistry, University of Konstanz, 78457 Konstanz, Germany*

*correspondence: burgess@tamu.edu

Content

Peptide synthesis 3

Circular Dichroism (CD) experiments 4

IR-detected T-jump dynamics 7

Experimental methods for T-jump IR experiments 9

Peptide characterization 10

References 19

Peptide synthesis

Peptide synthesis protocols below followed the procedures published before.[1]

Linear peptides: The linear control peptides were synthesized using standard Fmoc peptide synthesis protocols (Scheme S1) on TentaGel S RAM resin (capacity = 0.22 mmol/g) on a LibertyBlue peptide synthesizer. Couplings were carried out using 5 equivalents (equiv) of Fmoc-protected amino acid, 10 equiv of Oxyma, 10 equiv of DIC. Coupling reactions were allowed to proceed for 2 minutes in DMF at 90 °C, after which Fmoc deprotection was carried out with 20% (vol/vol) piperidine in DMF at 90 °C for 1 minute. Upon completion of the peptide sequence assembly on resin and deprotection of the final Fmoc group, the *N*-terminal amine was acetylated by treatment with 25% (vol) acetic anhydride and 3.5% (vol) DIPEA in DMF. Cleavage and deprotection from the solid support were carried out using TFA/H_2O/TIPS (95/2.5/2.5 vol/vol) for 3 hours at room temperature. The resin was then filtered and washed with excess TFA. The isolated TFA solution was then evaporated, and the peptide was precipitated by adding cold diethyl ether. The peptide suspension in ether was centrifuged to pellet followed by decanting the ether. The pellet was then washed twice with cold diethyl ether. Purification of the resulting peptides was achieved by high performance liquid chromatography (HPLC) on a reversed-phase C8 column to yield peptide with purity >95%. Purity and identity were assessed using ESI-MS and analytical HPLC on a C18 column.

Bicyclic *C*-capped peptides: The linear peptides were synthesized using standard Fmoc peptide synthesis protocols (Scheme S1) on TentaGel S RAM resin (capacity = 0.22 mmol/g) on a LibertyBlue peptide synthesizer. The three cystines were placed at *C3*, *Ccap* and *C”* near the *C*-terminus. Couplings were carried out using 5 equivalents (equiv) of Fmoc-protected amino acid, 10 equiv of Oxyma, 10 equiv of DIC. Coupling reactions were allowed to proceed for 2 minutes in DMF at 90 °C, after which Fmoc deprotection was carried out with 20% (vol/vol) piperidine in DMF at 90 °C for 1 minute. Upon completion of the peptide sequence assembly on resin and deprotection of the final Fmoc group, the N-terminal amine was acetylated by treatment with 25% (vol) acetic anhydride and 3.5% (vol) DIPEA in DMF. Cleavage and deprotection from the solid support were carried out using TFA/H_2O/TIPS (95/2.5/2.5 vol/vol) for 3 hours at room temperature. The resin was then filtered and washed with excess TFA. The isolated TFA solution was then evaporated, and the peptide was precipitated by adding cold diethyl ether. The peptide suspension in ether was centrifuged to pellet followed by decanting the ether. The pellet was then washed twice with cold diethyl ether and dissolved in 1:1 mixture of aqueous buffer (20mM NH_4HCO_3) and ACN to make final concentration around 1 mM. 1.5 equiv of TBMB was added and the solution was stirred for 15 min at room temperature. Purification of the resulting peptides was achieved by high performance liquid chromatography (HPLC) on a reversed-phase C8 column to yield peptide with purity >95%. Purity and identity were assessed using ESI-MS and analytical HPLC on a C18 column.

Bicyclic *N*-capped peptides: The linear peptides were synthesized using standard Fmoc peptide synthesis protocols (Scheme S1) on TentaGel S RAM resin (capacity = 0.22 mmol/g) on a LibertyBlue peptide synthesizer. The three cystines were placed at *N’*, *N3* and *N4* at the

N-terminus. Couplings were carried out using 5 equivalents (equiv) of Fmoc-protected amino acid, 10 equiv of Oxyma, 10 equiv of DIC. Coupling reactions were allowed to proceed for 2 minutes in DMF at 90 °C under microwave, after which Fmoc deprotection was carried out with 20% (vol/vol) piperidine in DMF at 90 °C for 1 minute. Upon completion of the peptide sequence assembly on resin and deprotection of the final Fmoc group, cleavage and deprotection from the solid support were carried out using TFA/H_2O/TIPS (95/2.5/2.5 vol/vol) for 3 hours at room temperature (longer time is required for peptides with Arg). The resin was then filtered and washed with excess TFA. The isolated TFA solution was then evaporated, and the peptide was precipitated by adding cold diethyl ether. The peptide suspension in ether was centrifuged to pellet followed by decanting the ether. The pellet was then washed twice with cold diethyl ether and dissolved in 1:1 mixture of aqueous buffer (20mM NH_4HCO_3) and ACN to make final concentration around 1 mM. 1.5 equiv of TBMB was added and the solution was stirred for 15 min at room temperature. Purification of the resulting peptides was achieved by high performance liquid chromatography (HPLC) on a reversed-phase C18 column to yield peptide with purity >95%. Purity and identity were assessed using ESI-MS and analytical HPLC on a C18 column.

Dual-capped peptides: The seven residues at the *C*-termini, including six residues forming Bicyclic *C*-cap, and the one before it, were first coupled on the resin, either manually or automatically using peptide synthesizer. Here Cys were in the form of Cys(Mmt). The Fmoc group was intact for the last residue, and then selective deprotection of Mmt followed by solid phase CLIPS was conducted to form Bicyclic *C*-cap on solid phase. Specifically, a solution of 1% TFA/DCM was prepared. 2 mL solution was added to the syringe, shaking for 2 min, then drained. This procedure was repeated, and the solutions normally would change from yellow, to orange, to red, then back to orange, and finally to light yellow after around 10 to 15 rounds of deprotections. After that, the beads are washed with several rounds of DCM and DMF. For solid phase cyclization, a 3 mL DMF solution with 2 eq. TBMB and 10 eq. DIPEA are prepared, then added to the syringe, and reacted for 1 hour, followed by thorough wash with DMF and DCM before the next step. After thorough wash by DCM and DMF, solid phase peptide synthesis was continued to grow the rest amino acids on the short *C*-terminal sequence. Cys at the *N*-termini were in the form of Cys(Trt). After final cleavage from resin and precipitation in ether, peptides were dissolved in solution for solution phase CLIPS to build Bicyclic *N*-cap. Purification of the resulting peptides was achieved by high performance liquid chromatography (HPLC) on a reversed-phase C18 column to yield peptide with purity >95%. Purity and identity were assessed using ESI-MS and analytical HPLC on a C18 column or LCMS.

Circular Dichroism (CD) experiments

Concentrations of the stock solution were determined by 288 nm absorption of Trp with ε = 5560 ($M^{-1}cm^{-1}$). Each sample was dissolved in PBS (pH 7.4) with the final concentration

at 10 μM. CD spectra were acquired using circular dichroism spectrometer (Chirascan) equipped with a temperature controller using 1 mm cell at a scan speed of 0.5 nm/sec at indicated temperature. Each sample was scanned three times and the averaged spectrum was smoothed.

Calculation of percent helicity: For simplicity, 'helicity' or 'helical' in the following text all refers to α-type. For each peptide, mean residue ellipticities ([θ]/(c*n), c = concentration of the peptide, n = number of amino acids in the peptide) were used to calculate % helicity, an empirical indicator of peptide helicity. Higher values of % helicity suggest the tested peptides are more helical. Percent helicity was calculated based on the equation: % helicity = $[\theta]_{222}/[\theta]_{max}$, where $[\theta]_{max}$ = (-44000 + 250T)(1 - k/n)] (n = number of amino acids in the peptide, T = 25.0 °C). According to Baldwin's work[2], k refers to the number of non-*H*-bonded peptide mainchain carbonyls in a carboxyamidated peptide; the residues with these carbonyls are less stabilized in helical conformations hence contribute less to $[\theta]_{222}$. For linear peptide, k is 3; for bicyclic *C*-capped peptides, k is 4; and for bicyclic *N*-capped peptides in this work, k is 5. For dual-capped system, k is 6 due to the sum of two caps. These are illustrated in Fig S1. The increment of k values in capped peptides is reasonable because forming caps which terminate helical propagation in one direction necessarily result in more non-helical residues that rarely contribute to $[\theta]_{222}$.

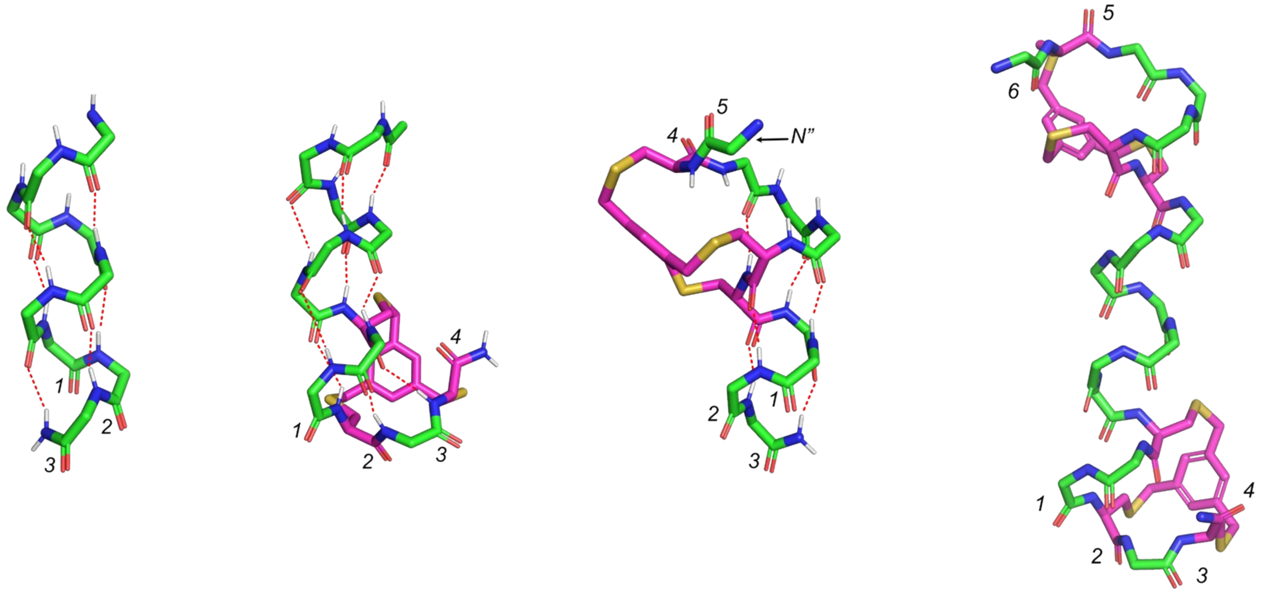

linear
k = 3

bicyclic *C*-cap
k = 4

bicyclic *N*-cap
k = 5

dual cap
k = 6

Figure S1: Non-*H*-bonded peptide mainchain carbonyls in different peptides. In ideal helical conformations, linear peptides have 3 non-*H*-bonded peptide carbonyls; bicyclic *C*-capped peptides have 4 (magenta represent three Cys and TMB segment); bicyclic *N*-capped peptides have 5; and dual-capped peptides have 6.

Variable Temperature CD Experiment: Each sample was dissolved in PBS buffer (pH 7.4) with the final concentration as 10 μM. CD spectra were acquired using circular dichroism spectrometer (Chirascan) equipped with a temperature controller using 1 mm cell at a scan

speed of 2 nm/sec at indicated temperature. Data point at each temperature was scanned three times and the averaged spectrum was smoothed. Temperature range (°C) between 277.6 to 367.0 K with a step of 5 was used in the study. Mean residue ellipticity were calculated and used to make graphics.

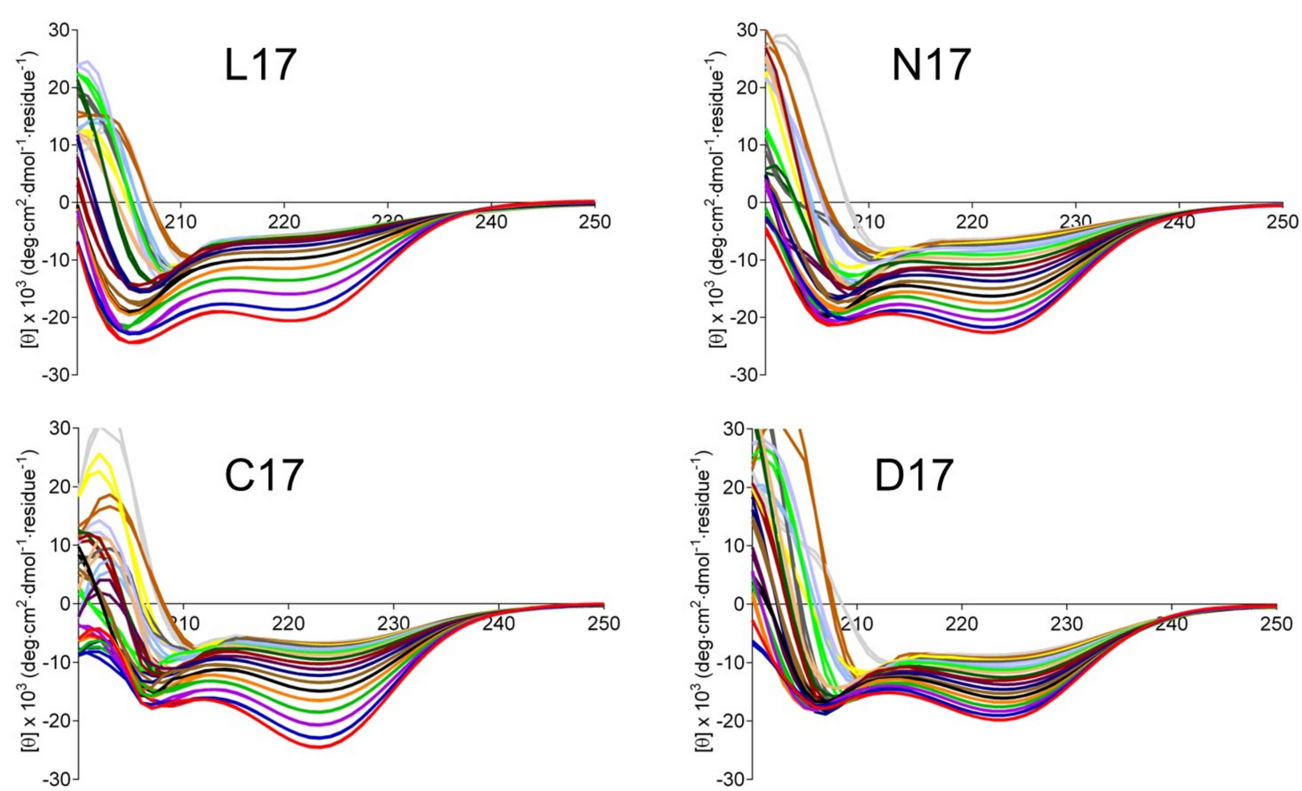

Figure S2: Smoothed CD curves of L17, N17, C17 and D17 from 277.6 to 367.0 K at 10 μM in PBS buffer.

Fitting of θ_{222} from VT-CD experiments to deduce thermodynamic parameters: The calculations follow equations in previous publication[3]:

The equations below assume a two-state transition of a monomer from a folded to unfolded state, and the heat capacity of the folded and unfolded states are equal.

[Parameters]

h=-20000 ;starting enthalpy in cal/mol

tm=50 ;starting TM (observed) in °C.

u=-32000; mean residue ellipticity of 100% folded helical protein

l=-5000; mean residue ellipticity of unfolded protein

[Variables]

v=col(1) ; temperature in °C

w=col(2) ; mean residue ellipticity, absorbance, etc

[Equations]

m=tm+273.15 ; convert Centigrade to Kelvin

t=v+273.15; convert Centigrade to Kelvin

k=exp((h/(1.987*t))*((t/m)-1)); calculate folding constant at any given temperature

y=k/(1+k); calculate fraction folded at any given temperature

f=((u-l)*y)+ l ; calculate ellipticity at any given temperature

fit f to w; fit the calculated ellipticity to the observed ellipticity.

The fitting was conducted in Graphpad Prism 6 using least square fit. Fitting R^2 for peptides are 0.9996 (L17), 0.9998 (N17), 0.9996 (C17), and 0.9996 (D17), indicating overall good fit. Thermodynamic parameters including enthalpy (H) and median temperature (T_m) were deduced from the fit, and entropy was calculated by equation $\Delta S = \Delta H/T_m$.

IR-detected T-jump dynamics

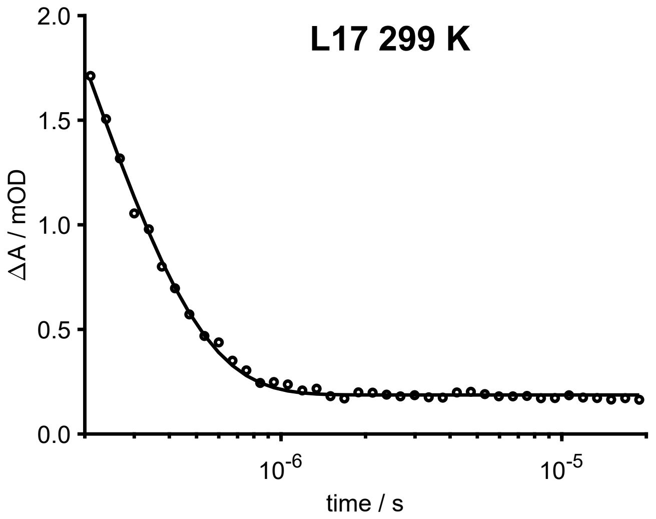

Figure S3: T-jump relaxation dynamics of L17. Transient at a final temperature of 299 K measured at 1630 cm^{-1} with monoexponential fit in the range 200 ns to 20 µs. The first data point of the transient starts at 200 ns after the T-jump due to experimental limitations.

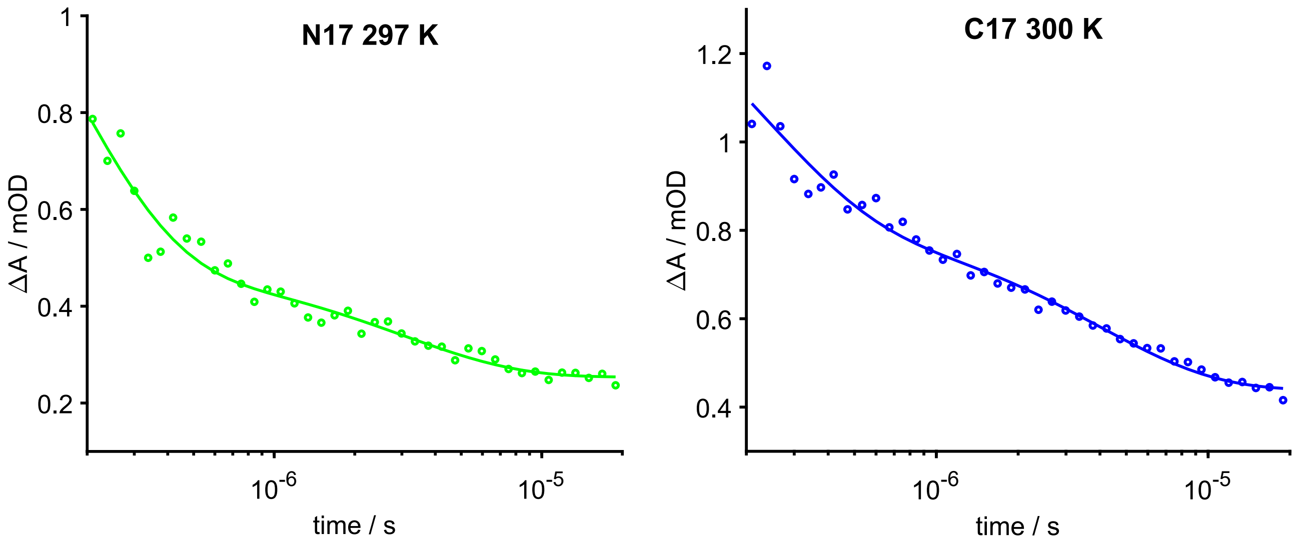

Figure S4: T-jump relaxation dynamics of N17 and C17. Transients at final temperatures of 297 resp. 300 K measured at 1632 resp. 1628 cm^{-1} and fitted with biexponential function, showing the data are best represented with two time constants, a fast τ_f, in the range of hundreds of nanoseconds, and a slow τ_s, in the single digit microsecond range.

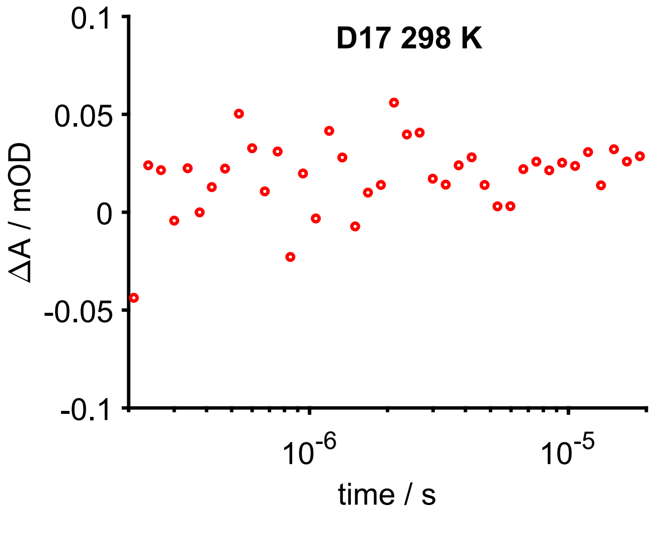

Figure S5: T-jump relaxation dynamics of D17. Transient at a final temperature of 298 K measured at 1630 cm^{-1}. Since the change in absorbance is pretty small, the fit results show large errors and resulting data are less reliable or even fail to converge, as is the case here.

Experimental methods for T-jump IR experiments

Sample preparation for IR-spectroscopy

To eliminate spectral interference from trifluoroacetate (TFA) counterions remaining from the peptide synthesis, peptides were dissolved in 0.1 M DCl and lyophilized three times. Afterwards the peptides were adjusted to a pD of ~3.7 with 0.1 M NaOD and lyophilized again. IR samples were prepared in D_2O with a concentration of ~5 mg/mL at pD ~3.7 (for D17 ~20 mg/mL in D_2O with 20% DMSO) and were placed in demountable cells consisting of CaF_2 windows separated by a Teflon spacer with 100 µm optical pathlength.

Time-resolved IR-detected temperature-jump dynamics

T-jump IR measurements were performed using the quantum cascade laser-based (QCL) spectrometer that has been described in detail previously [4]. Relaxation dynamics of the peptides were probed at selected wavenumbers with a quantum cascade laser (QCL), installed in a MIRcat-QT laser system (Daylight Solutions Inc., USA). The continuous-wave (cw) QCL used (M2062- PCX) had a tuning range from 1730 to 1480 cm^{-1}. The rapid temperature jump is initiated by a 10 ns pulse at 2090 nm of a Ho:YAG laser (IPG Photonics Corporation, USA), exciting a solvent (D_2O) vibration, thereby rapidly increasing the peptide temperature. The pump pulse is split into two counter propagating parts; one of the pump pulses is delayed by 5 ns to produce a homogenous heating profile in the sample cell preventing cavitation effects [5]. The initial temperature of the sample was controlled by a water bath connected to the sample holder. The temperature jump was calculated by referencing the measured absorbance change of the solvent to the corresponding temperature- dependent FITR spectra of D_2O [4]. A T-jump magnitude of ~ 5 – 7 K was used for the experiments. At each temperature about 1000 transients were averaged in two independent measurement series. To exclude distorted transients caused by cavitation effects, a self-developed software filter (MATLAB2021a, The MathWorks, USA) was applied to save the most reliable data after collection. To account for solvent kinetics, both solvent and peptide sample were measured sequentially. The solvent-only signal was scaled appropriately and subtracted from the peptide sample signal. The resulting transients were subjected to a quasi-logarithmic averaging procedure so that an equal number of points were distributed in each time decade (20 points per decade) leading to a significant reduction of noise and distortion signals. Measurements of the set of 17-mers were performed with the same samples as for the equilibrium FTIR measurements. The relaxation times were evaluated in a time interval from 200 ns up to 20 µs using a monoexponential decay at a broad range of final temperatures:

$$y(t) = y_0 + Ae^{-\frac{t}{\tau}} \qquad (eq. S1)$$

where y_0 is an offset, A is the pre-exponential factor and τ is the relaxation time.

For the both monocapped variants C17 and N17 biexponential fits delivered much better fits than monoexponentials:

$$y(t) = y_0 + A_f e^{-\frac{t}{\tau_f}} + A_s e^{-\frac{t}{\tau_s}} \qquad (eq. S2)$$

where τ_f is the relaxation time of the faster dynamic and τ_s of the slower one with related preexponential factors A_f and A_s. Also triexponential fits were tried out but did not show any further improvements. Fitting procedures were performed with Origin 2021b (OriginLab Corporation, USA) software.

Peptide characterization

L17: Ac-AKAAAAKAAAAKAAAAW

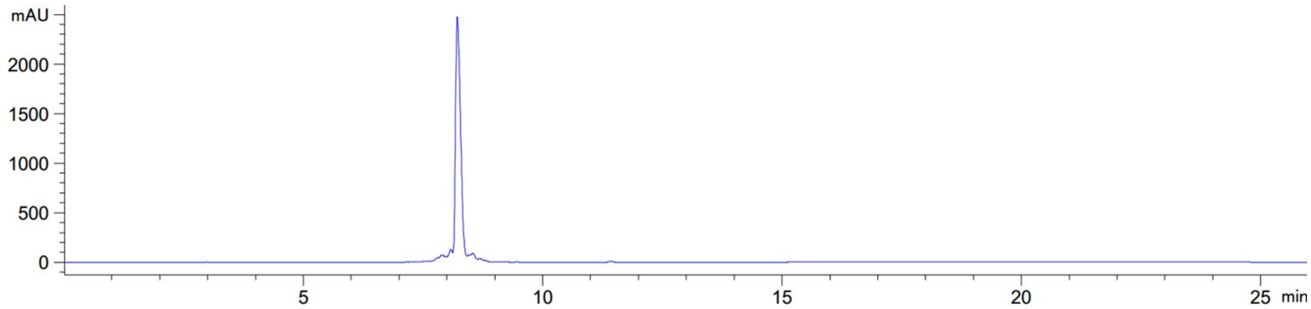

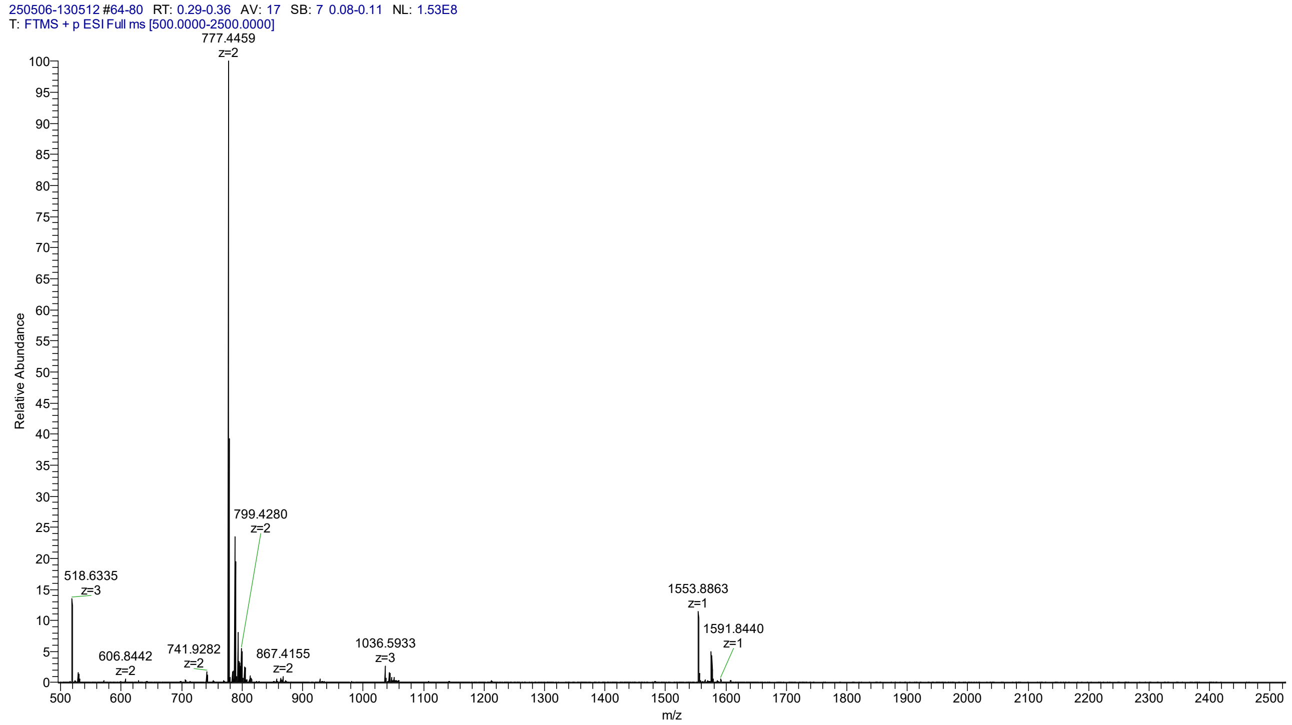

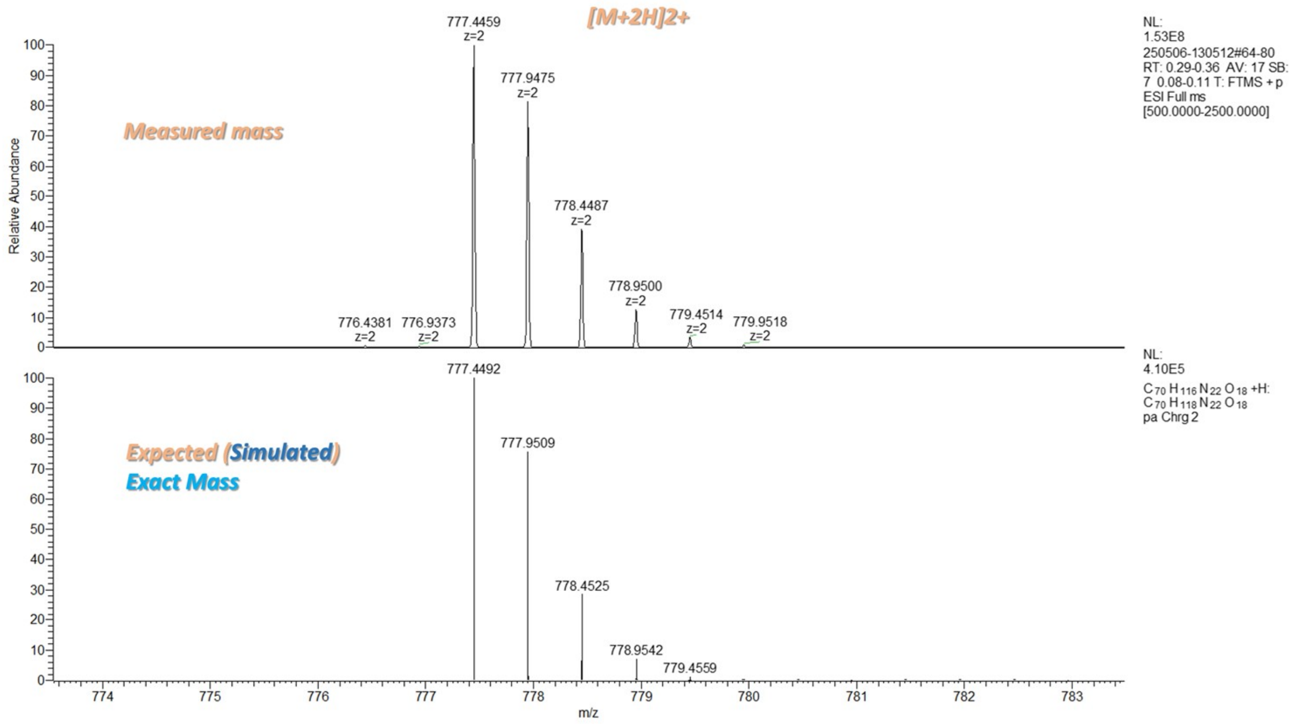

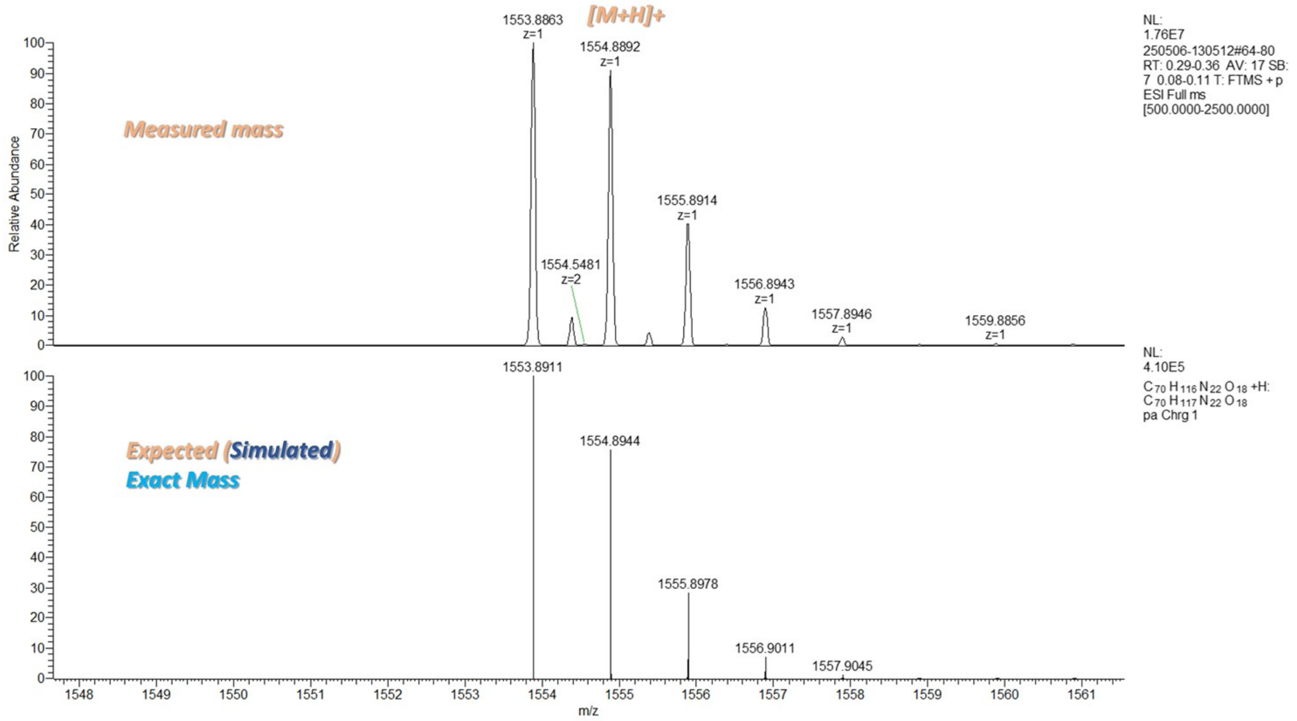

exact mass calculated: 1552.88; MS detected: 1553.8863 (M+H), 777.4459 (M+2H)

C17: Ac-AAAAKAAAAKW[CAACGC]$_{cyc}$

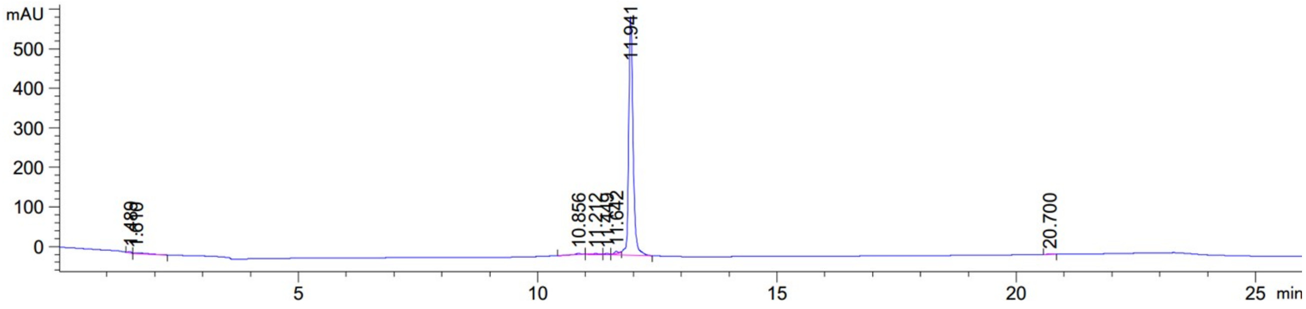

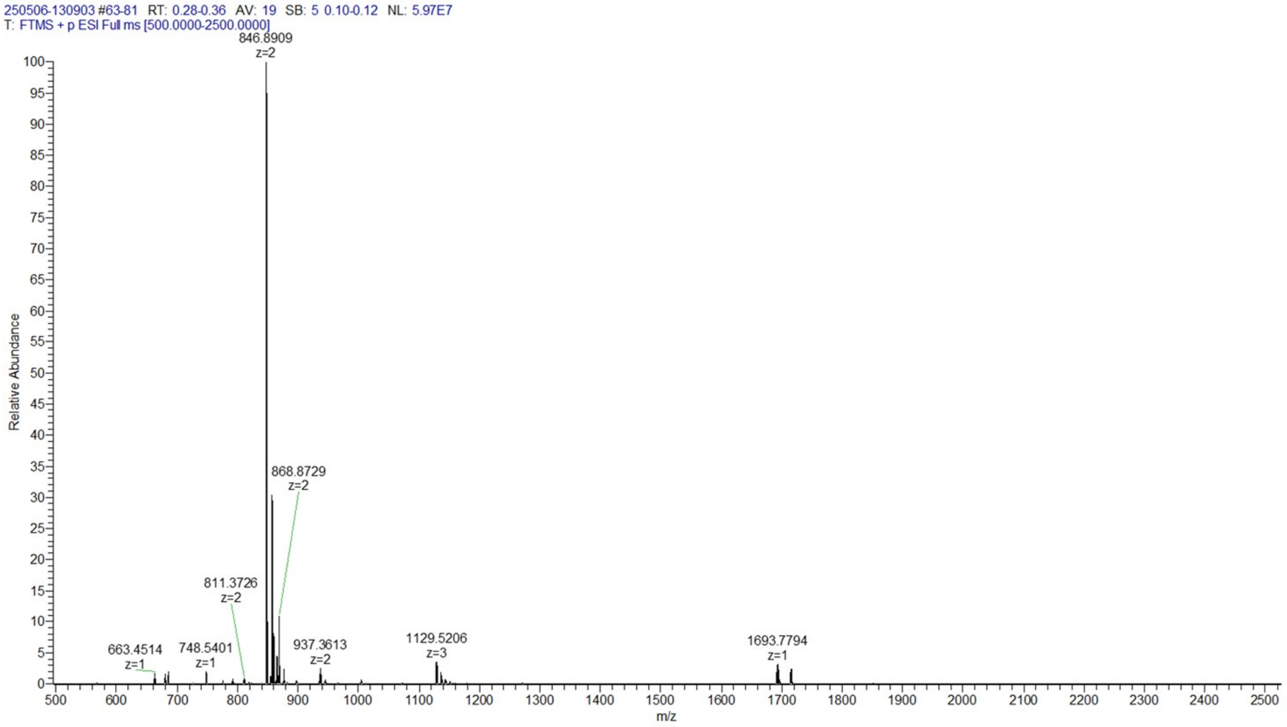

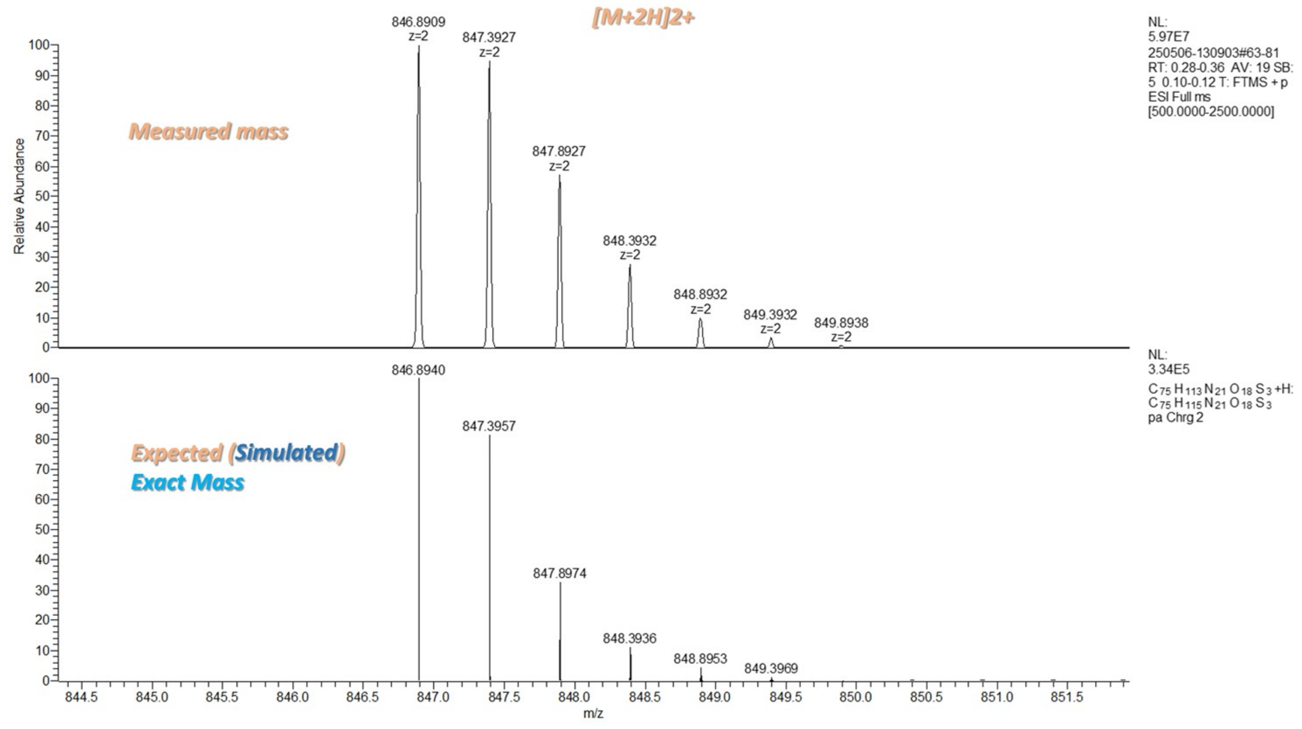

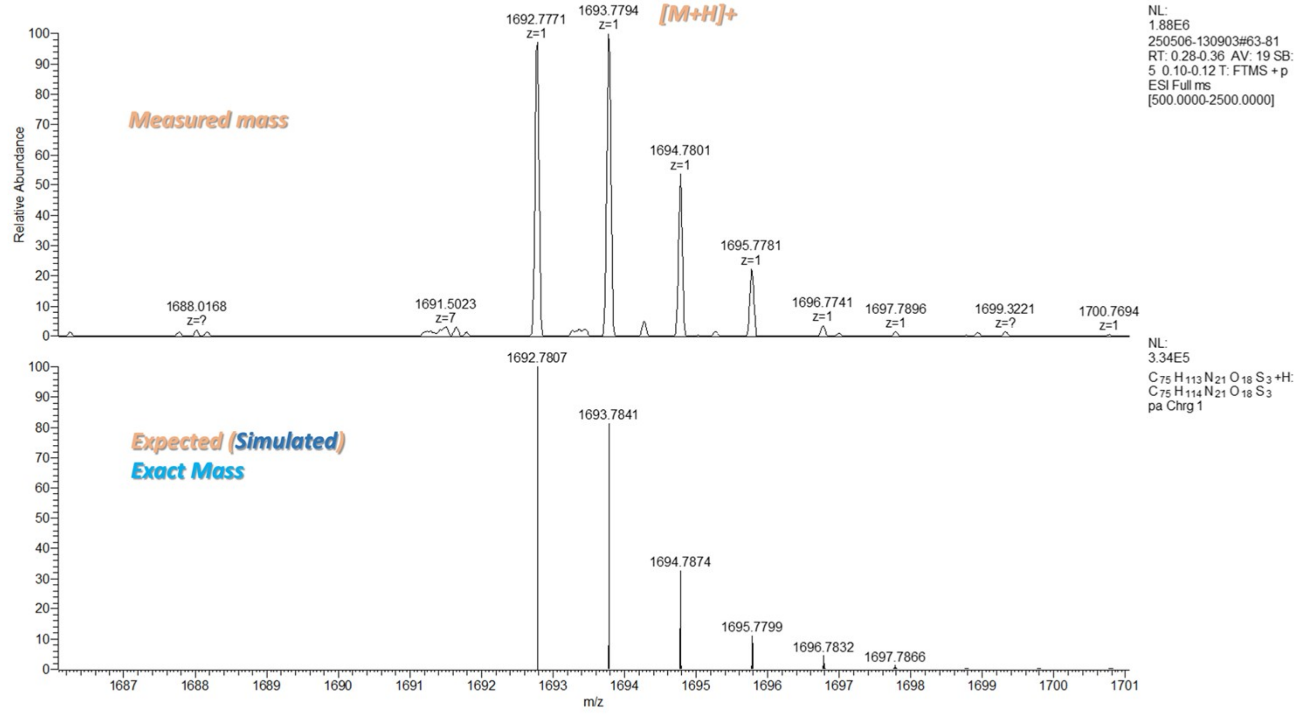

exact mass calculated: 1691.77; MS detected: 1692.7771 (M+H), 846.8909 (M+2H)

N17: W[CDAACC]$_{cyc}$AAAKAAAAKA

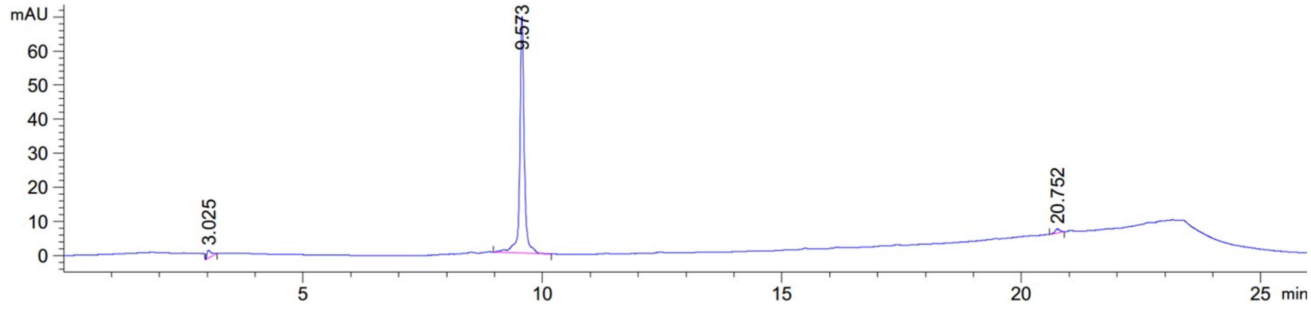

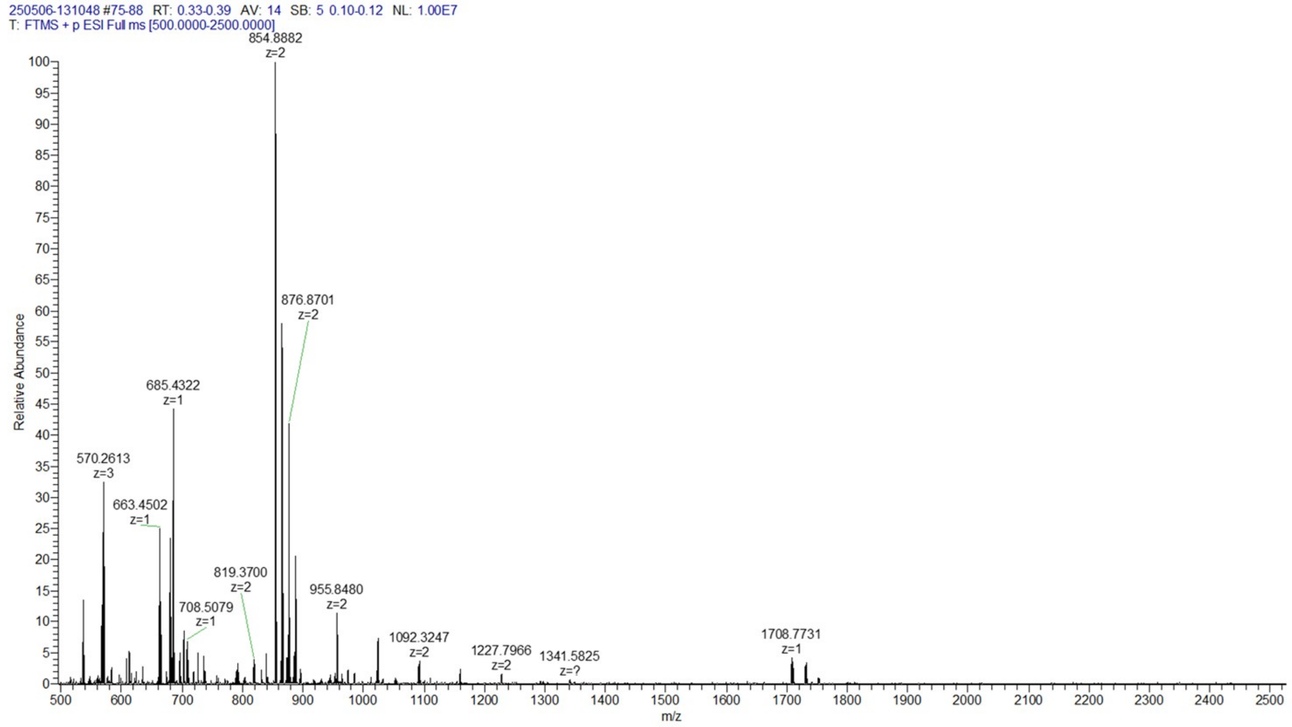

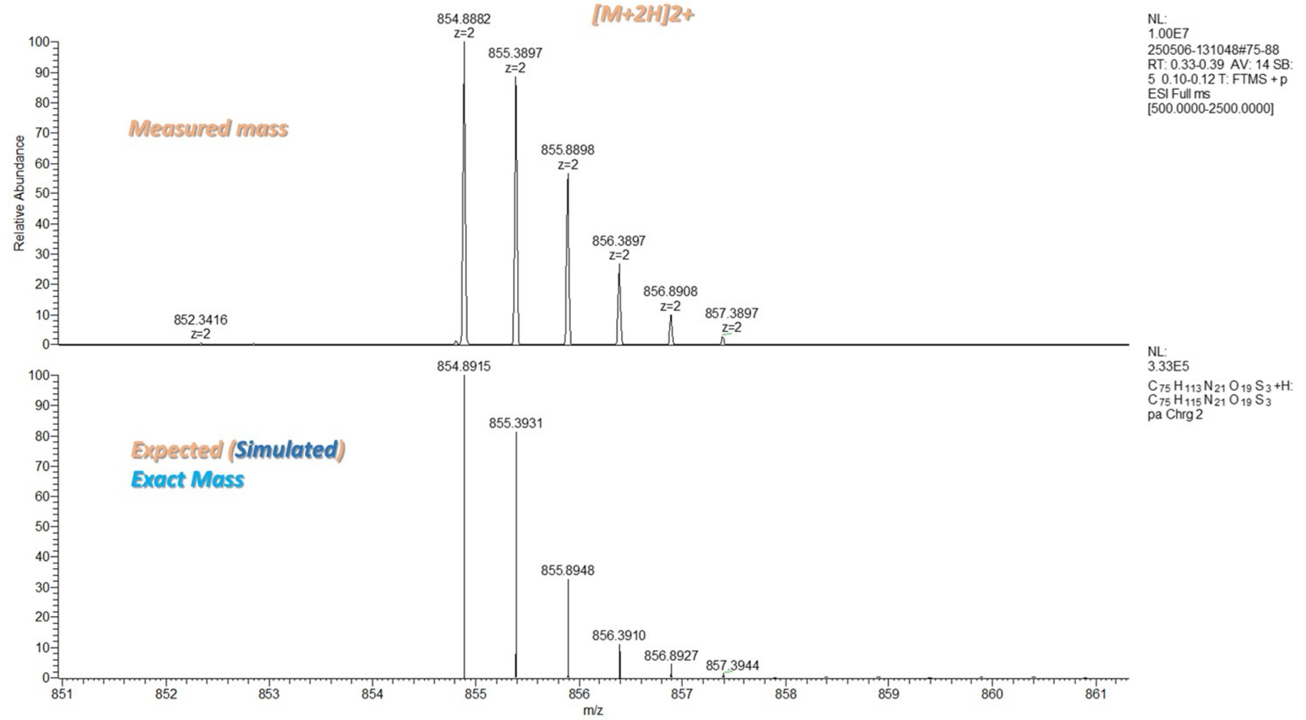

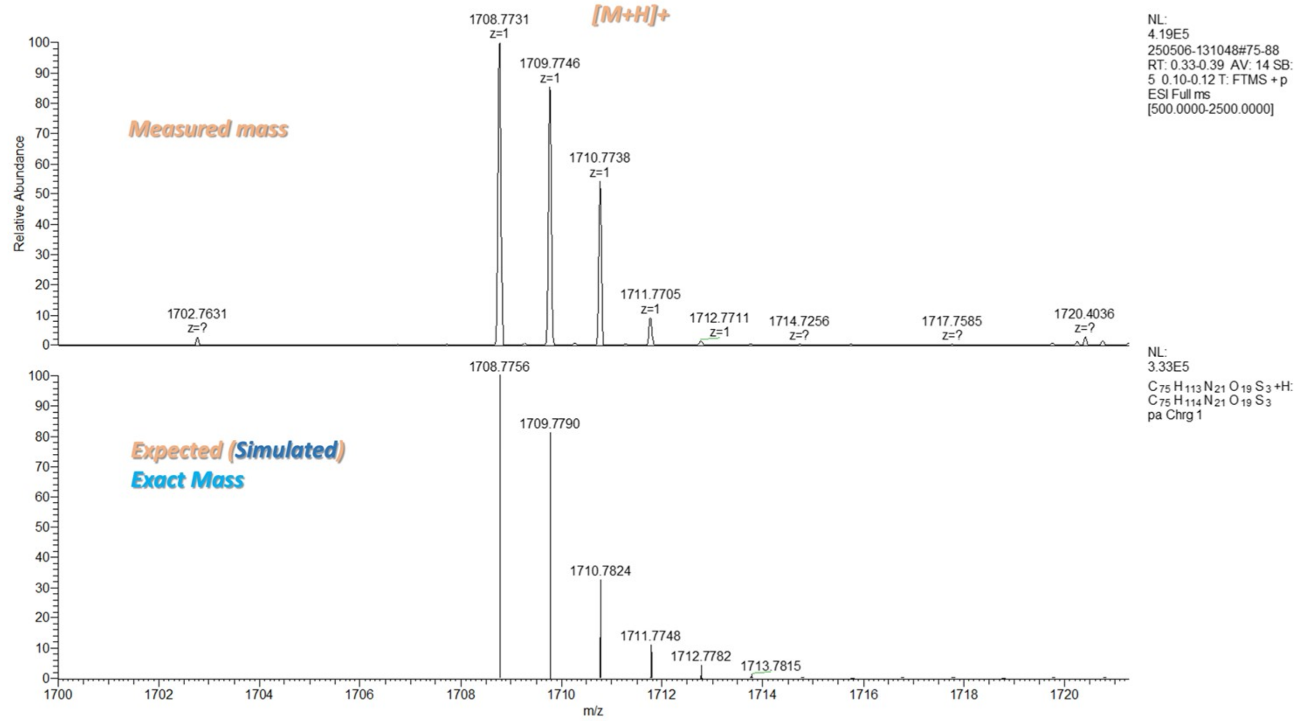

exact mass calculated: 1707.77; MS detected: 1708.7731 (M+H), 854.8882 (M+2H)

D17: W[CDAACC]$_{cyc}$AKAA[CAACGC]$_{cyc}$

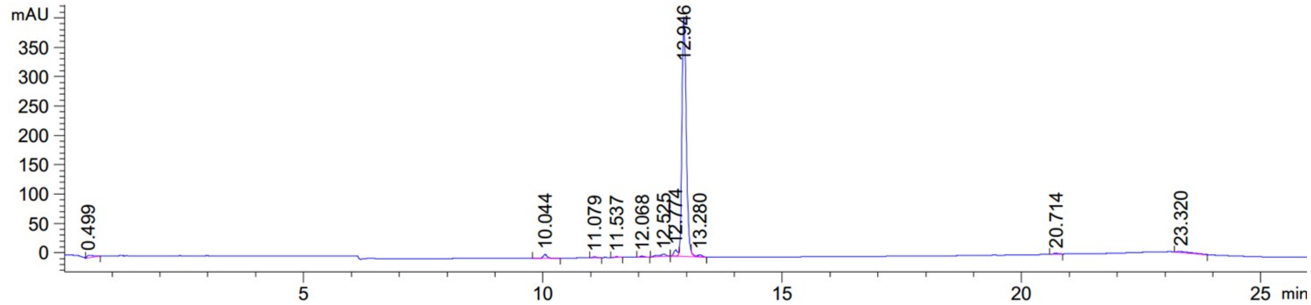

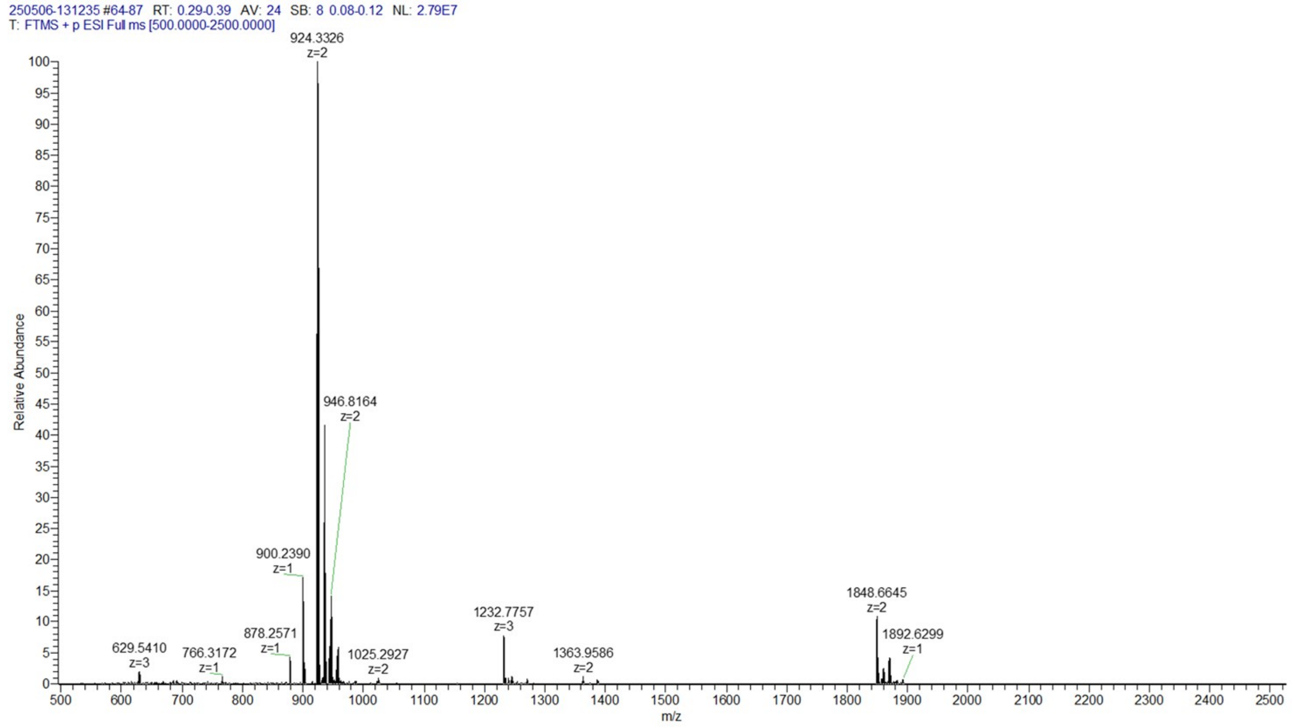

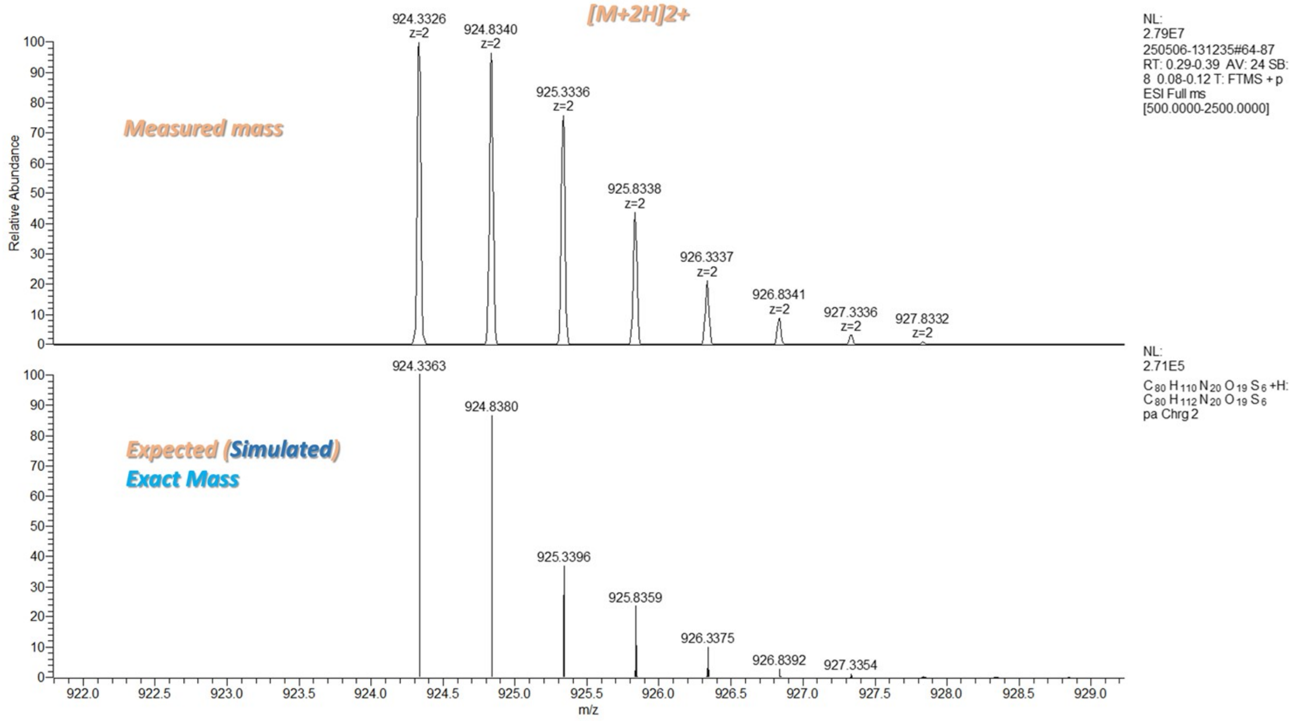

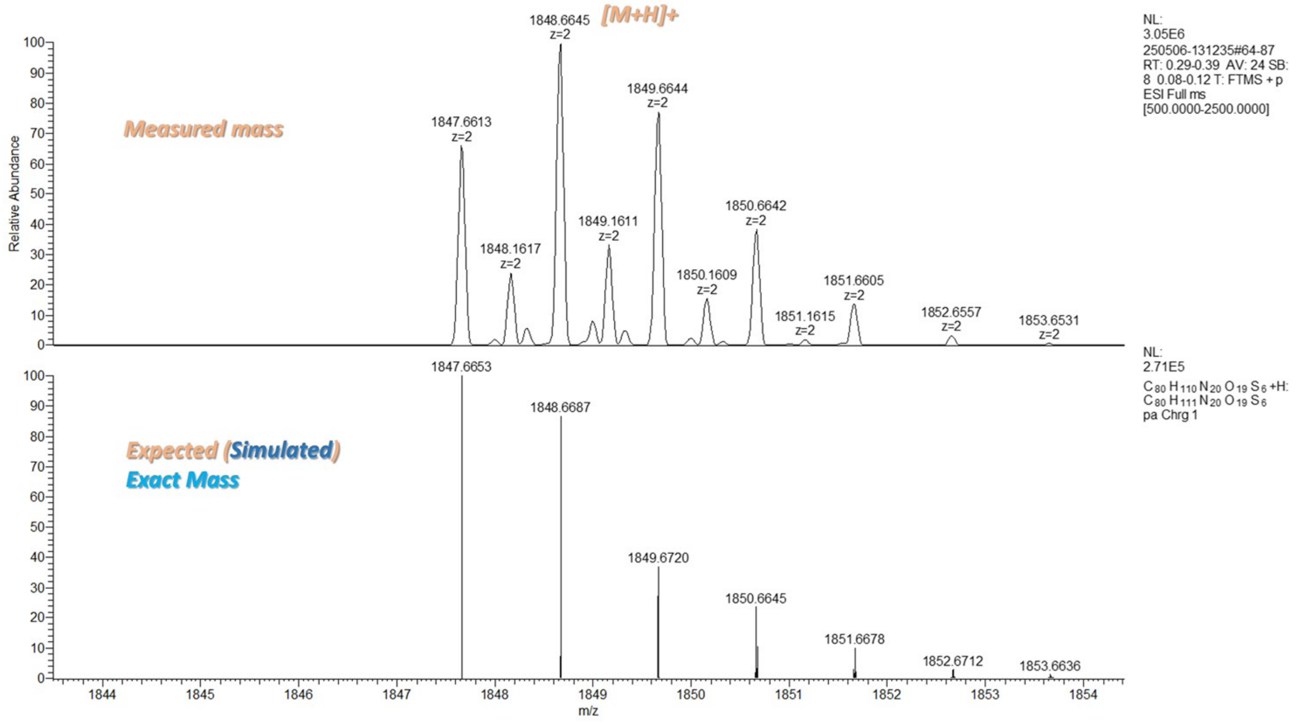

exact mass calculated: 1846.66; MS detected: 1847.6613 (M+H), 924.3326 (M+2H)

D15: W[CDAACC]$_{cyc}$AK[CAACGC]$_{cyc}$

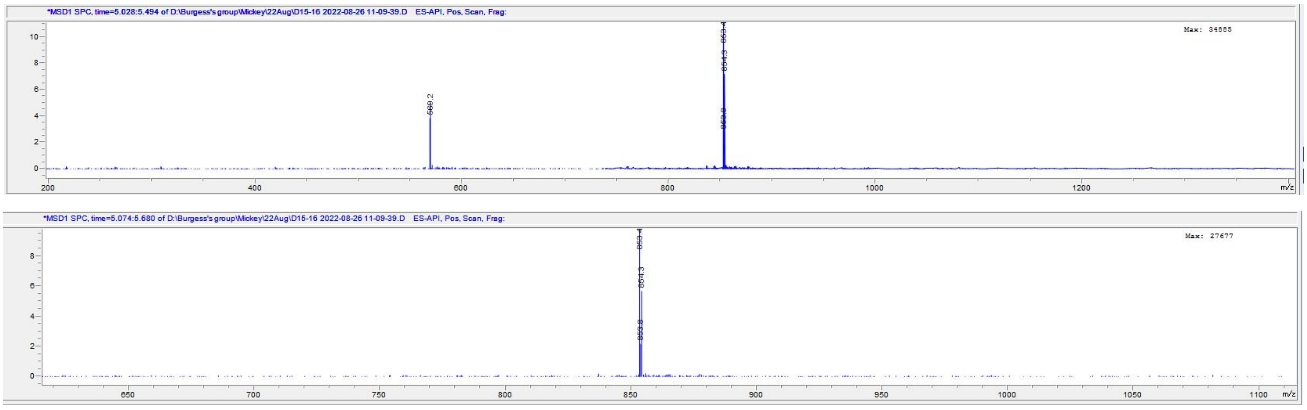

exact mass calculated: 1704.58; MS detected: 853.4 (M+2H), 569.2 (M+3H)

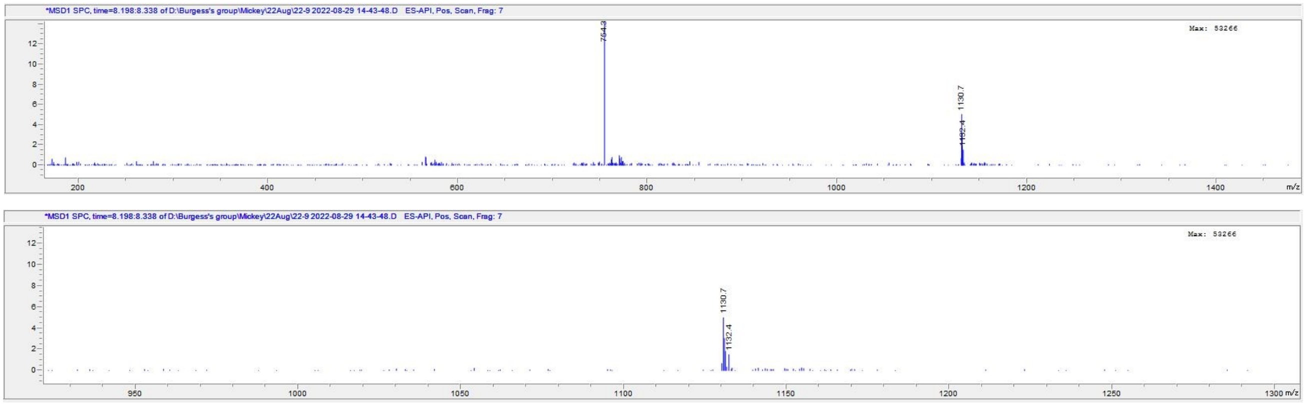

D22: W[CDAACC]$_{cyc}$AKAAAKAAA[CAACGC]$_{cyc}$

exact mass calculated: 2259.91; MS detected: 1130.7 (M+2H), 754.3 (M+3H)

References

[1] T. Mi, Z. Gao, Z. Mituta, K. Burgess, *J. Am. Chem. Soc.* **2024**, *146*, 10331-10341.

[2] a P. Luo, R. Baldwin, *Biochemistry* **1997**, *36*, 8413–8421; b C. Rohl, R. Baldwin, **2004**, *295*, 1-26.

[3] N. Greenfield, *Nat. Protoc.* **2006**, *1*, 2527-2535.

[4] a H. Siu, K. Hauser, *JPhysChemLett* **2022**, *13*, 4543-4548; b D. Scheerer, H. Chi, D. McElheny, T. A. Keiderling, K. Hauser, *Chemistry* **2020**, *26*, 3524-3534; c A. Popp, D. Scheerer, H. Chi, T. A. Keiderling, K. Hauser, *ChemPhysChem* **2016**, *17*, 1273-1280; d A. S. Popp, D. Scheerer, B. Heck, K. Hauser, *Spectrochim. Acta A Mol. Biomol. Spectrosc.* **2017**, *181*, 192-199.

[5] W. O. Wray, T. Aida, R. B. Dyer, *Appl. Phys. B* **2002**, *74*, 57-66.
